# Comorbidity of Auditory Processing, Attention, and Memory in Children With Word Reading Difficulties

**DOI:** 10.3389/fpsyg.2019.02383

**Published:** 2019-10-22

**Authors:** Rakshita Gokula, Mridula Sharma, Linda Cupples, Joaquin T. Valderrama

**Affiliations:** ^1^Department of Linguistics, Macquarie University, Sydney, NSW, Australia; ^2^HEARing Cooperative Research Centre, Melbourne, VIC, Australia; ^3^Centre for Language Sciences, Macquarie University, Sydney, NSW, Australia; ^4^National Acoustic Laboratories, Sydney, NSW, Australia

**Keywords:** word reading difficulty, auditory processing, cognition, digit memory, receptive language

## Abstract

**Objectives:**

To document the auditory processing, visual attention, digit memory, phonological processing, and receptive language abilities of individual children with identified word reading difficulties.

**Design:**

Twenty-five children with word reading difficulties and 28 control children with good word reading skills participated. All children were aged between 8 and 11 years, with normal hearing sensitivity and typical non-verbal intelligence. Both groups of children completed a test battery designed to assess their auditory processing, visual attention, digit memory, phonological processing, and receptive language.

**Results:**

When compared to children who were good readers, children with word reading difficulties obtained significantly lower average scores on tests of auditory processing, including the frequency pattern test, gaps in noise, frequency discrimination, Dichotic Digit difference Test, and Listening in Spatialized Noise. The two groups did not differ on the discrimination measures of sinusoidal amplitude modulation or iterated rippled noise. The results from children with word reading difficulties showed that 5 children (20%) had comorbid deficits in auditory processing, visual attention, and backward digit memory; whereas 12 children (48%) had comorbid auditory processing and visual attention deficits only, and 2 children (8%) had comorbid deficits in auditory processing and digit memory; the remaining children had only auditory processing, visual attention, or digit memory deficits.

**Conclusion:**

The current study highlights the general co-existence of auditory processing, memory, and visual attention deficits in children with word reading difficulties. It is also noteworthy, however, that only one fifth of the current cohort had deficits across all measured tasks. Hence, our results also show the significant individual variability inherent in children with word reading difficulties.

## Introduction

Co-morbidities in children with developmental disorders are more the norm than the exception. Several studies have reported that children with auditory processing difficulties have coexisting deficits in language skills (Benasich et al., 2002; McArthur and Bishop, 2004; Wible et al., 2005; Sharma et al., 2009), attention skills (Dhamani et al., 2013; Allen and Allan, 2014; Gyldenkærne et al., 2014; Sharma et al., 2014; Tomlin et al., 2015), and/or memory skills (Allen and Allan, 2014; Sharma et al., 2014). Other studies suggest that children with reading difficulties exhibit coexisting deficits in auditory processing (Goswami et al., 2002; Banai and Ahissar, 2004; Fischer and Hartnegg, 2004; Halliday and Bishop, 2006a; Sharma et al., 2006, 2009; Iliadou et al., 2009; Reid et al., 2010; Hämäläinen et al., 2012), language skills (Scarborough, 1990; Wise et al., 2007; Scarborough et al., 2009) attention skills (Willcutt and Pennington, 2000; Willcutt et al., 2005) and/or working memory abilities (Swanson et al., 1989, 2009; Swanson, 1993). Considering the weight of evidence to date, which shows that children are more likely to have deficits across multiple skills than deficits in isolated skills, this research was designed to investigate the range and frequency of different co-morbidities evident in children with word reading difficulties. Word reading difficulties were defined as scores that fell 1.5 standard deviation (SD) or more below the typical mean in oral reading of non-words (Badian, 1996; Compton et al., 2001; Banai and Ahissar, 2006; Paul et al., 2006; Baird et al., 2011).

The variables of auditory processing, attention, working memory, phonological processing, and language were measured in the search for co-morbidities, due to their frequently observed association with reading ability. The clinical motivation for this study lay in the belief that targeting multiple functional areas in the assessment process would significantly help a clinician to collect sufficient information to guide a multi-disciplinary approach in order to manage the full range of deficits that a given child may exhibit.

### Auditory Processing and Reading

Auditory processing has been studied extensively in relation to children’s reading ability, with the earliest theory proposing an association between auditory processing and phonic decoding skills in particular; that is, the ability to read by mapping letters onto sounds (Tallal, 1980, 1984). Tallal (1980) demonstrated this association using an auditory temporal-order judgment task in which children with reading difficulty made significantly more errors than children with typical reading skill at fast but not slow presentation rates.

According to Ramus (2001), however, sensory theories of dyslexia, such as that proposed by Tallal and colleagues, suffered from a number of potential weaknesses, including: the failure of some studies to find an association between reading and auditory processing; the finding that only a subgroup of participants is often responsible for reported group differences; and the possibility that apparent sensory deficits might instead reflect differences in the strategies used for task completion. Furthermore, Ramus questioned whether there was sufficient evidence to support claims of a causal association between perceptual processing and word reading indirectly through a potential phonological relationship.

Despite such criticisms, there is continued interest in the possible role of basic auditory processing deficits as they relate to reading difficulty (e.g., Goswami et al., 2002; Leppänen et al., 2010; Goswami, 2011; Huss et al., 2011; Casini et al., 2018). Rise-time theory, described originally by Goswami et al. (2002), proposed that children with dyslexia experience a basic auditory processing deficit that interferes with their perception of the rhythmic timing of speech. In support of this proposal, they used a beat detection task to show that children with reading difficulties performed significantly more poorly than control children who were matched on chronological age. More recently, Casini et al. (2018) assessed the temporal and intensity discrimination skills of children with poor word reading. They reported that children with word reading deficits performed more poorly on a temporal discrimination task than a group of peers with typical reading skills who were matched on chronological age. The groups did not differ significantly, however, in their judgments of intensity. In one of the few longitudinal studies in this area, Leppänen et al. (2010) reported that auditory ERPs in neonates were correlated with measures of phonological awareness at 3.5 years of age and letter knowledge at 5 years of age, and were significant predictors of 9-year-old measures of reading speed and reading accuracy after controlling for a range of other potentially important variables.

These various findings support a role for basic auditory processes in the development of typical reading skills, but other researchers have questioned such a role. For example, Snowling et al. (2018) found no evidence in their longitudinal data for an association between early frequency discrimination (measured at 4.5 and 5.5 years of age) and later reading outcomes (measured at 5.5 and 8 years old). Furthermore, because executive function at 4.5 predicted frequency discrimination at 5.5, they suggested that poor performance on auditory processing tasks might be due to comorbid attentional difficulties in some children. This suggestion accords with the hypothesis offered by Ramus (2001, p. 395) that auditory processing deficits might be found only in people with reading difficulty who also have some other developmental disorder, which he refers to as a “hidden factor.” It is also consistent with Leppänen et al.’s (2010) suggestion that an early auditory processing deficit may not be sufficient, on its own, to cause a reading difficulty. In sum, despite decades of work in the field of auditory processing and reading, the evidence of specific auditory processing skills and their contribution to reading is not well understood. A question of direct relevance to research endeavors in this field is how auditory processing should be measured.

### Relevant Auditory Processing Skills

Auditory processing is an umbrella term that encapsulates abilities such as auditory discrimination (e.g., frequency discrimination), spectral resolution and discrimination (e.g., amplitude and frequency modulation), temporal ordering (e.g., frequency patterning), and performance in degraded listening conditions (e.g., listening in noise) (American Speech-Language-Hearing Association [ASHA], 1996). While there are more recent definitions of auditory processing offered in the literature, none define the specific skills and co-morbidities of auditory processing as explicitly as the (American Speech-Language-Hearing Association [ASHA], 1996, 2005) documentation (Jerger and Musiek, 2000).

Ramus (2001) and Goswami (2015) in their respective reviews of the literature noted that most theories of reading difficulty that attribute an important role to auditory processing deficits assume an intervening association with phonology; that is, auditory processing deficits result in phonological impairment, which in turn leads to a reading difficulty. As noted above, however, auditory processing can be measured in a multitude of ways. Missing from the literature is a detailed understanding of how and why different measures of auditory processing may be associated with particular components of phonological processing and/or reading subskills.

Another challenge for the field is the presence of ambivalent results across a number of auditory processing tasks. For instance, Hämäläinen et al. (2012) in their review reported that children with reading difficulties performed significantly worse than children with no reading difficulties in frequency discrimination (FD; Banai and Ahissar, 2006; Goswami et al., 2010). Conversely, a study by Adlard and Hazan (1998) reported that FD was unaffected in children with reading difficulties. In the review (Hämäläinen et al., 2012), there were studies that reported significantly worse thresholds on frequency modulation (FM) at slow rates of 2 Hz in children with reading difficulties compared to children with no reading difficulties (Gibson et al., 2006; Dawes et al., 2009; Wright and Conlon, 2009). In contrast, other researchers found no differences in FM thresholds of children with reading difficulties and their age-matched peers at modulation rates of 2 Hz (Halliday and Bishop, 2006b; Dawes et al., 2009), 20 Hz (Halliday and Bishop, 2006b), or 240 Hz (Adlard and Hazan, 1998). Similar dichotomous reports have been noted for amplitude modulated (AM) thresholds as well. For instance, Rocheron et al. (2002) reported significant group differences for very low and high modulation rates of 4 and 128 Hz; whereas Hämäläinen et al. (2009) reported no significant differences on the same task, at a modulation rate of 20 Hz.

Findings from studies that assessed children’s performance on the more commonly used clinical tests such as Frequency Pattern Test (FPT), Dichotic Digit Test (DDT), Gaps in Noise (GIN), and speech in noise (Sharma et al., 2006; Iliadou et al., 2009) are more consistent in showing that children with reading difficulties have poorer responses than their age-matched peers with typical reading skills. Barker et al. (2017) used an iPad-based app to assess FPT and DDT and found that children with poor reading comprehension were significantly worse on both measures compared to children with good reading comprehension skills.

The different patterns of results obtained across various studies that involve similar tasks and children of a similar age are of interest, because they raise questions about the reliability of tests used (e.g., test–retest), heterogeneous characteristics of the population, and variability in performance (e.g., intrinsic attention during assessment). The theoretical basis for the contribution of auditory processing to reading will remain a challenge while these three aspects remain unanswered. Therefore, a secondary aim of the current study was to evaluate the individual profiles of a sample of children with word reading difficulties on well-established auditory processing tasks.

### Phonological Processing, Vocabulary, Visual Attention, Digit Memory and Reading

The relationship between phonological processing and word reading is well established, and therefore the current study included assessment of phonological processes to confirm the presence of individual variability, if any, in the current cohort (Wagner and Torgesen, 1987). For the same reason, assessments of receptive vocabulary, visual attention, and digit memory were included in the test battery. Notably, however, the aim of the current study was not to determine *whether* these skills were worse in our cohort of poor word readers than in a peer group of typical readers, but rather to document significant group differences and to discover the extent to which the current sample of children with word reading deficits exhibited individual variability in these skills.

Reading involves not only the conversion of print to speech, but also the assignment of meaning to words and larger units of language, with comprehension being the ultimate intention (Ouellette, 2006). Regardless of whether the relationship between reading and receptive vocabulary is direct (Scarborough et al., 2009), or one that is mediated by phonological awareness (Whitehurst and Lonigan, 1998), children’s vocabulary is an essential component of oral language that is crucial for skilled reading (Perfetti et al., 1996; Muter et al., 2004; Ouellette, 2006). Vocabulary growth appears to play a role in the development of phoneme awareness (Metsala, 1999; Goswami, 2001; Walley et al., 2003), which in turn is associated with word decoding. Felton et al. (1987) found that children with reading difficulties were significantly poorer on measures of receptive vocabulary than age-matched controls with typical reading skills. Ouellette (2006), in a study of 60 children, found vocabulary to be the sole measure to concurrently predict decoding ability (measured using oral reading of non-words) when variables of age and non-verbal intelligence were controlled.

The cognitive measures of particular relevance to this study are those of visual attention and digit memory. Visual attention, especially in the spatial domain, is employed for reading (Stevens and Bavelier, 2012). Casco et al. (1998) studied the association between visual selective attention and reading rate in children. Visual selective attention was assessed using a task that required children to identify target alphabets that were interleaved amidst visually similar symbols. Children who made more errors on the visual attention task were significantly slower readers. Rabiner and Coie (2000) reported that attention difficulties predicted the concurrent reading achievement of typically developing children, after measures such as IQ, and prior reading achievement were controlled within the group.

Working memory is another cognitive domain that has been studied extensively to assess its association with children’s reading skill. Working memory, as described by Baddeley (1986), includes a central executive component which monitors the phonological loop and the visuo-spatial sketchpad which are responsible for sound based input and visual input respectively. Working memory tasks assess an individual’s ability to maintain task-related information while processing that information further or performing another cognitive task. Swanson et al. (2009) in a meta-analysis of 88 studies noted poorer working memory, as measured using reading/listening span, digit span, and digits backwards, in children with reading disabilities in support of Baddeley’s theoretical framework.

### The Current Study: Reading, Auditory Processing, Phonological Processing, Visual Attention, Digit Memory and Language Skills

The current research had two aims. The first aim was to determine whether children with word reading difficulties have poorer auditory processing skills than age-matched control children with typical reading skills. The second aim was to identify individual profiles and commonly occurring co-morbidities within the group of poor non-word readers. In order to determine the individual profiles, all 25 children were tested on auditory processing, phonological processing, visual attention, digit memory, and language tasks. Before determining the profiles, the question of performance criteria was considered.

#### Performance Criteria

A crucial methodological question in any research that involves the identification of impaired performance is: what constitutes a deficit. A common strategy is to identify a deficit in terms of how far below the typical mean an individual score falls in standard deviation (SD) units. Wilson and Arnott (2013) discussed the impact of choosing a criterion for identification of auditory processing disorder, where the use of 1 SD below or 2 SD below the mean can lead to disparity in the numbers of children diagnosed. Notably, Wilson and Arnott chose to use minus 2 SD as the diagnostic criterion in line with ASHA guidelines. In accordance with this approach, a deficit in auditory processing was identified in the current study when individual scores fell 2 SD or more below the mean. A similar criterion was not deemed appropriate for all measures, however. Much of the cognitive literature is consistent in using 1 SD below the mean as the demarcation for deficits in attention (Dawes et al., 2009; Landerl and Willburger, 2010; Franceschini et al., 2012) and memory (Swanson et al., 1989, 2009; Swanson, 1993). The reading literature is different again, typically using 1.5 SD below the mean to indicate a deficit (Badian, 1996; Compton et al., 2001; Banai and Ahissar, 2006; Paul et al., 2006; Baird et al., 2011). The current paper is not designed to determine the most appropriate criteria for identification of atypically poor performance across the range of skills measured, and is therefore aligned with the published literature in defining deficits as follows:

• ≥2 SD below the mean for auditory processing tasks (American Speech-Language-Hearing Association [ASHA], 1996);
• ≥1.5 SD below the mean for reading;
• ≥1 SD below the mean for attention and memory.

We hypothesized that a majority of the children with word reading difficulties in the current study would have concurrent comorbidities on all of the measured skills.

## Materials and Methods

### Participants

Fifty-three children aged 8–11 years (Mean age in years ± SD = 9.7 ± 1.17) participated in the current study. Of the 53 children, 25 (9.5 ± 1.16 years) were identified as having reading concerns (henceforth referred to as word reading difficulty) because they scored at least 1.5 SD below the mean on the Castles and Coltheart 2 (CC2) test of non-word reading. Twenty-eight were typically developing control children (10.0 ± 1.09 years) who scored within 1 SD of the mean or better on the same test of non-word reading. The participants in the study did not report with any other developmental concerns. All participants spoke English as their first language and attended schools that used English as the medium of instruction. Participants were recruited through advertisements in the Macquarie University Speech and Hearing Clinic (Sydney, NSW, Australia), on social media sites, and in children’s magazines available freely to families across Sydney. Parents provided written informed consent for their children to participate in the study, and each child gave verbal assent as per the requirement of the Human Research Ethics Committee at Macquarie University (Reference No: 5201600441). Families received a token of appreciation for participating in the study. The study conformed, at all times, to the guidelines of the Australian Government: National Health and Medical Research Council (2018).

### Inclusion Criteria

All participants were tested for normal hearing sensitivity using clinical tests: otoscopy, tympanometry, pure tone audiometry, acoustic reflex thresholds, and distortion product otoacoustic emissions (DPOAEs). Acoustic reflex thresholds and DPOAEs were used to identify any underlying hearing loss that may not be picked up by audiometry. Non-verbal cognitive ability was also assessed to ensure that all children had an age-appropriate Non-Verbal Intelligence Quotient (NVIQ) of 85 or greater.

Otoscopy was conducted to determine the general health of the ear canal and identify any visible signs of infection. Tympanometry was carried out with a 226 Hz probe tone to test middle ear status. Pure tone audiometry (PTA) was conducted using the modified Hughson-Westlake procedure (5 dB steps). During PTA, participants were instructed to indicate whenever they heard a sound and were asked to pay close attention to soft sounds. PTA and tympanometry were carried out in a sound-treated booth. Children were included in the study if their hearing thresholds were ≤15 dB HL at octave frequencies between 250 Hz and 8000 Hz (American National Standards Institute [ANSI], 1996) and showed normal ear compliance and ear canal volume (Medwetsky et al., 2009).

Acoustic reflex thresholds were obtained through ipsilateral and contralateral stimulation at octave frequencies from 500 to 4000 Hz. Children were included in the study if ipsilateral and contralateral acoustic reflexes were detected at 500, 1000, and 2000 Hz consistent with their audiometric thresholds. DPOAE testing was conducted for both ears between 1000 and 6000 Hz. Children were included if they had present DPOAEs on three consecutive frequencies with at least a signal to noise ratio (SNR) of +6 dB (Medwetsky et al., 2009) consistent with PTA and immittance results.

The Wechsler Non-Verbal Scale of Ability (WNV) was used to assess non-verbal cognitive ability (Wechsler and Naglieri, 2006). This test includes a non-verbal mode of instruction using pictures from the test manual. The matrices and spatial span subtests of the WNV assessed the children’s non-verbal reasoning and spatial memory skills.

In the matrices subtest, children finished an incomplete matrix of images by pointing to the correct image from within a list of options. The test items gradually increased in difficulty. Testing stopped once a child responded incorrectly to four out of five consecutive matrices. In the spatial span task, children were presented with blocks on a board. Each block had a number visible only to the examiner. The examiner pointed to a specific number of blocks in a given sequence that the child had to imitate either in the same order or in reverse order. The test began with three blocks. For each length of block number, two sequences were presented. If the child was able to imitate one or both sequences for a given number of blocks, the number of blocks presented increased by one. Testing stopped once a child responded incorrectly to both sequences at a given block length. A standard score, equivalent to NVIQ, was assigned on the basis of the raw scores for matrices and spatial span subtests (Wechsler and Naglieri, 2006). A standard score of ≥85 (Massa and Rivera, 2009) was required for a child to be included in the study.

**FIGURE 1:**
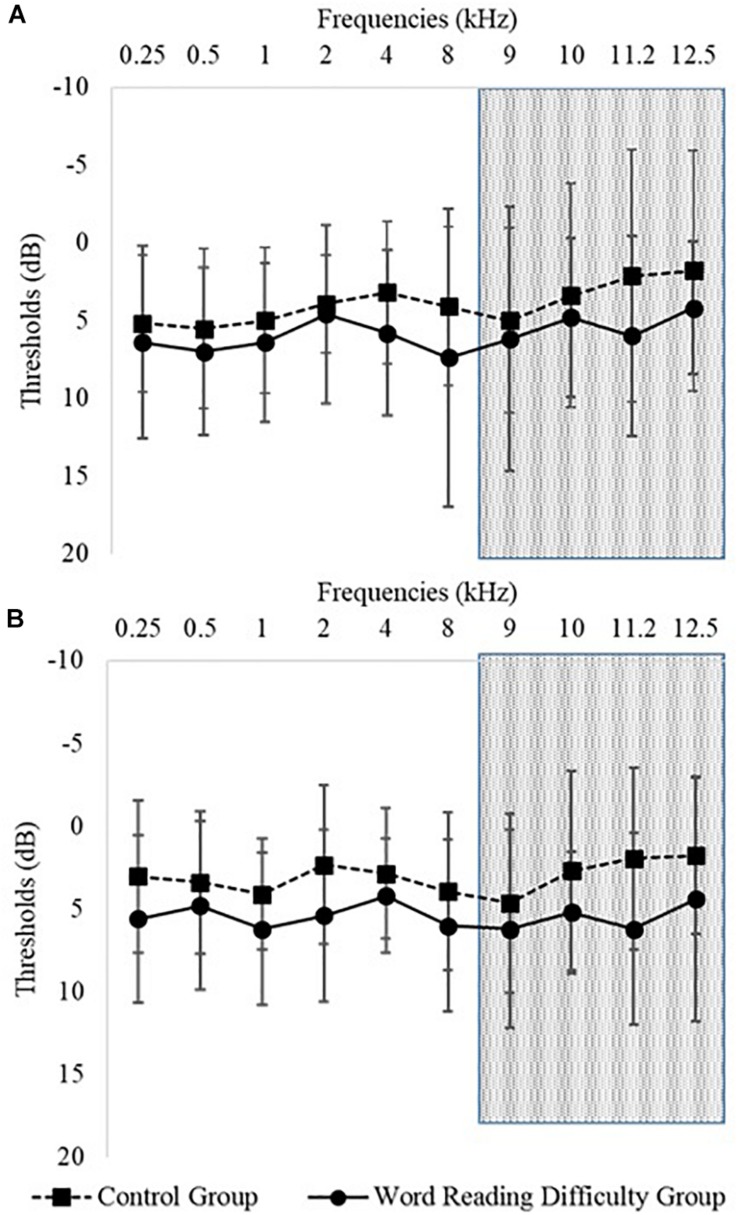
Mean hearing thresholds according to the groups: the figure displays the mean thresholds (and standard deviations in error bars) for the children from the two groups (squares are for control group and circles for the word reading difficulty group) between 250 to 12.5 kHz. **(A)** Represents the thresholds of the two groups for their right ear and **(B)** represents thresholds for the left ear. The shaded represents the demarcation between clinically assessed frequencies and extended high-frequency thresholds.

Table 1 presents the means and SDs of children’s age, PTA, and WNV scores according to group. This table shows no significant difference for the audiometry thresholds obtained by children in the two groups from 500 to 4k Hz for both ears [*F*(3,153) = 1.64, *p* = 0.18]. The group mean audiometry result for the left and right ear is presented in Figure 1 that shows no significant difference for the extended high frequencies 8k to 12.5k Hz [*F*(4,204) = 0.35, *p* = 0.82]. The control group showed a small but significant advantage on the WNV when compared to the group with word reading difficulties [*F*(1,51) = 5.28, *p* = 0.03], but both groups scored around 1 SD above the mean on average (see Table 1).

**TABLE 1:** Means (and SDs in parentheses) for age, PTA (0.5 – 4 kHz), and WNV scores for children in the two groups.

**Groups**	**Age^∗^ (*SD*)**	**PTA^∗∗^ Right (*SD*)**	**PTA^∗∗^ Left (*SD*)**	**WNV (*SD*)**
Control, *N* = 28	10.0 (11)	4.4 (3.4)	3.2 (3.0)	118.7 (10.4)
Females, *N* = 11	9.3 (0.9)	4.2 (3.9)	3.4 (3.3)	119.2 (10.7)
Males, *N* = 17	10.5 (1.0)	4.4 (3.2)	3.0 (3.0)	118.4 (10.5)
Word Reading Difficulty, *N* = 25	9.4 (1.2)	5.6 (4.7)	4.8 (4.0)	112.4 (9.29)
Females, *N* = 9	9.8 (1.2)	5.3 (4.3)	4.6 (4.8)	113.1 (4.96)
Males, *N* = 16	9.4 (1.2)	6.3 (4.6)	5.4 (3.3)	112.1 (11.2)

*^∗^Age is presented in years. ^∗∗^PTA is presented in dB HL.*

### Tests

Testing for each participant occurred over two separate days within a 7- to 10-day period. Each testing session lasted 2.5–3 h with regular breaks. Testing was conducted in a distraction-free Macquarie University Speech and Hearing Clinic room (Sydney, NSW, Australia). To minimize any effect of procedural bias, testing order was randomized.

The Maximum Likelihood Procedure (MLP) toolbox (Grassi and Soranzo, 2009) was used to develop the FD (Peter et al., 2014), sinusoidal amplitude modulation (SAM; Peter et al., 2014), and iterated ripple noise (IRN; Peter et al., 2014) tests for the study. The stimuli for the behavioral hearing tests (FD, SAM, and IRN threshold) were created at a sampling rate of 44100 Hz. Staircase method was used for threshold estimation. The clinically available test stimuli for FPT (Musiek, 2002) and GIN (Shinn et al., 2009) were routed through a clinical audiometer (AC 40) and played through HDA 200 headphones (Sennheiser Electronic Corporation, Old Lyme, CT, United States) at a level of 50 dB HL (American National Standards Institute [ANSI], 1996). Listening in Spatialized Noise-Sentences (LiSN-S; Cameron and Dillon, 2007) and Dichotic Digit difference Test (DDdT; Cameron et al., 2016) were played through the computer via commercially available software, through headphones accompanying the LiSN-S test (HD 215, Sennheiser Electronic Corporation, Old Lyme, CT, United States).

Calibration of the auditory stimuli (FPT, SAM, IRN, FD, and GIN) was carried out using a Type 2231 sound level meter (SLM), a Type 4152 artificial ear, a Type 4144 1-inch pressure microphone, and an AC 40 Audiometer (Brüel & Kjaer Sound & Vibration Measurements A/S, Naerum, Denmark). The stimuli were calibrated to ensure that the output from the Audiometer was 50 dB HL.

Auditory processing assessment for all children included FPT – right and left ear (Musiek, 2002), GIN – right and left ear (Shinn et al., 2009), FD (Peter et al., 2014), SAM – 4 and 40 Hz (Peter et al., 2014), IRN – 32 and 4 iterations (Peter et al., 2014), LiSN-S (Cameron and Dillon, 2007) and Dichotic Digit difference Test – dichotic and diotic listening (DDdT; Cameron et al., 2016). Phonological processing was assessed using the elision subtest from the Comprehensive Test of Phonological Processing (Wagner et al., 1999). Visual attention was assessed using the sky search, map mission, creature counting, and same and opposite world subtests from the Test of Everyday Attention for Children (Manly et al., 2001). Digit memory was assessed using the digit forward and backward subtask from Clinical Evaluation of Language Fundamentals – Fourth edition (CELF-4; Semel et al., 2006). Receptive vocabulary was assessed using the Peabody Picture Vocabulary Test – Fourth edition (PPVT-4; Dunn and Dunn, 2007). The current study used tests that have extensively been employed in previous research. The details of the tests are provided in Supplementary Tables 1, 2 to enable reduplication of the current research. The current study also used three auditory processing measures: FD, IRN, and SAM, the stimulus and methodology for which, tend to vary across publications. Details of these tests are presented in Table 2.

**TABLE 2:** Details of the auditory processing tests employed in the current study.

**Measures**	**Tests**	**Procedure**
Auditory processing	Frequency Discrimination (FD)	*Stimuli:* A 1000 Hz tone was used as the stimulus. The target tone was varied between 1100 and 1001 Hz by multiplicative step size factors of 2 for the first two reversals and 1.41 thereafter (Moore et al., 2011). The stimuli were 250 ms in duration. There was a 50 ms gap between the two tones. *Procedure:* FD is a measure affected by learning, and changes with repetition of the task (McArthur and Hogben, 2012). Therefore, the current study incorporated testing across two runs. Thresholds were estimated using a 3 Alternative Forced Choice (AFC) task which uses the 2-down 1-up tracking method which helps calculate the 70.7% correct point on a psychometric function (Levitt, 1971). The target tone was provided in the first, second, or third interval, alongside two comparison tones. Feedback was provided after each trial to inform the participant whether their response was correct or incorrect. *Response and Scoring:* The participants were asked to identify the sound that was different amongst the three stimuli. Every correct response resulted in a reduction in the difference between the tones. Every wrong response led to an increase in the difference between the tones. The arithmetic mean of the last six reversals in a block of 12 was taken as the threshold. A logarithmic transformation was applied to the threshold. The norms for the test were taken from Halliday and Bishop (2006a). Two such responses were obtained from the participants across separate blocks. As per Moore et al. (2008), assessment of track widths across two response blocks of the participants ensured “genuine good” and “genuine poor” responses compared to non-compliant responses from children who lost their attention during the task. Visual assessment of the track widths for each participant ensured that the study did not include any participants with non-compliant responses due to poor attention. A lower score (in Hz) represented better frequency discrimination ability in a participant.
	Threshold for detection of Sinusoidal Amplitude Modulation (SAM)	*Stimuli:* For the SAM task white noise modulated at 4 and 40 Hz was used. Over the first two reversals, the modulation depth was reduced by 2 dB after which the reversals were in 1 dB step size till the threshold was estimated. *Procedure:* Similar to the procedure employed to obtain the FD score. *Response and Scoring:* Similar to the response and scoring in the FD test. No logarithmic transformation was applied to the thresholds. The norms for the test were taken from Peter et al. (2014). Two such responses were obtained from the participants across separate blocks. A lower score (in dB) represented better thresholds for detection of AM in a sinusoidal signal.
	Threshold for discrimination of Iterated Rippled Noise (IRN)	*Stimuli:* IRN with 4 and 32 iterations were created with a delay of 10 ms. Four iterations correspond to a weak pitch percept, and 32 iterations have a strong pitch percept (Krishnan et al., 2014). A 500 ms Gaussian noise was low-pass filtered at 3000 Hz and added back to itself to create the IRN stimulus. The starting level for the gain (in dB) was set at 9.8 dB, which corresponds to a gain of 0.32 (Peter et al., 2014). From the starting level, the gain was reduced in step sizes of −3 dB (for the first two responses) and −1.5 dB (over the course of the rest of the test). *Procedure:* Similar to the procedure for the FD task. *Response and Scoring:* Similar to the response and scoring in the FD task. No logarithmic transformation was applied to the thresholds. The norms for the test were taken from Peter et al. (2014). Two such responses were obtained from the participants across separate blocks. A lower score (in dB) represented better thresholds for detection of pitch embedded in the IRN signal.
		

### Statistical Analysis

One-way Analyses of Variance (ANOVAs) were used to compare the groups’ performance on each task. A conservative *p*-value of 0.01 was used to reduce the likelihood of type I errors associated with multiple comparisons. Standard scores were not available for all auditory processing measures; in these cases, Analyses of Covariance (ANCOVAs) were conducted on raw scores, with age as a covariate to account for any age-related differences in performance.

### Criteria for Individual Sub-Profiles

Word and non-word reading abilities were assessed using the CC2 test. This test includes three word lists containing regular words (whose correct pronunciation is in line with letter-sound rules; e.g., take), irregular words (whose correct pronunciation conflicts with letter-sound rules; e.g., eye), and non-words (e.g., norf). The children in the current study were assessed on their ability to read all three types of words, and those whose non-word reading scores fell 1.5 SD or more below the normative mean were classified as having word reading difficulties. In accordance with the performance criteria outlined earlier (see section Performance Criteria), children in the study were identified as having an auditory processing deficit if they scored 2 SD or more below the mean on one or more of the auditory processing tests (American Speech-Language-Hearing Association [ASHA], 1996). They were identified as having poor attention if they scored at least 1 SD below the mean on either of the visual attention tasks – selective or switching, and they were identified as having a working memory problem if their performance on the backwards digit task was at least 1 SD below the typical mean. Correlations were also conducted across the tests used in the current study to observe the linear relationships between the variables across which the groups were compared.

## Results

### Tests of Reading and Phonological Processing

Children’s raw scores for regular, irregular and non-word reading on the CC2 test were compared to the age-based norms to derive *z*-scores. The control group achieved mean *z*-scores of 1.7 (*SD* = 0.85), 0.97 (*SD* = 0.78), and 1.1 (*SD* = 0.91) on regular word, irregular word, and non-word reading, respectively. By contrast, the group with word reading difficulties had mean *z*-scores of −1.9 (*SD* = 0.40), −1.4 (*SD* = 0.66), and −1.9 (*SD* = 0.40) respectively. Children in the control group achieved standard scores of 13.8 (*SD* = 1.20) on the phonological awareness test of elision while the children with word reading difficulties had an average standard score of 10.3 (*SD* = 2.65) with a statistically significant difference between the groups (*F*[1,51] = 45.0, *p* < 0.001).

### Auditory Processing Tests

Scores on most of the individual auditory processing tasks were significantly worse, on average, for children with word reading difficulty than for the control group (see results from univariate analyses in Table 3). The group differences were significant for FD, FPT, GIN, DDdT scores (dichotic and diotic), and LiSN-S measures (low cue; high cue); but there were no significant group effects for SAM thresholds or IRN thresholds.

**TABLE 3:** ANOVA results alongside the means (and standard deviations in parentheses) across the two groups for the individual auditory processing tests.

**Test**		**Control Mean (*SD*)**	**Word Reading Difficulty Mean (*SD*)**	***F*-value**	***p*-value**	**Effect size**
FD	Run 1 (log)	1.09 (0.31)	1.72 (0.53)	24.6	< 0.001^∗^	0.330
	Run 2 (log)	1.00 (0.30)	1.59 (0.59)	18.24	< 0.001^∗^	0.267
IRN	32 iterations	19.20 (2.81)	18.67 (2.73)	0.34	0.561	0.007
	04 iterations	13.28 (3.10)	11.62 (2.43)	5.13	0.028	0.093
SAM	40 Hz	−15.78(1.59)	−14.71(3.50)	1.13	0.291	0.022
	4 Hz	−11.96(2.57)	−9.55(3.79)	5.31	0.025	0.096
FPT	Right (%)	93.78 (6.95)	71.82 (21.26)	23.06	< 0.001^∗^	0.316
	Left (%)	92.23 (11.12)	70.10 (22.51)	18.10	< 0.001^∗^	0.266
GIN	Right (ms)	4.96 (0.88)	6.36 (1.93)	9.14	0.004	0.155
	Left (ms)	5.14 (0.80)	6.36 (1.93)	7.04	0.010	0.124
DDdT	Dichotic (*z*-score)	0.62 (1.14)	−1.02(1.17)	26.86	< 0.001^∗^	0.345
	Diotic (*z*-score)	0.59 (0.88)	−1.29(1.25)	40.50	< 0.001^∗^	0.443
LiSN– S	Low cue Score (*z*-score)	−0.43(0.81)	−1.25(0.98)	11.13	0.002	0.179
	High cue Score (*z*-score)	0.42 (0.74)	−0.29(0.89)	10.36	0.002	0.169
	Talker advantage (*z*-score)	−0.18(0.88)	−0.30(0.96)	0.23	0.634	0.004
	Spatial advantage (*z*-score)	0.02 (1.27)	−0.44(0.99)	2.19	0.148	0.041
	Total advantage (*z*-score)	0.73 (0.79)	0.29 (0.83)	3.91	0.053	0.071

*In these analyses, the df, error df for the *F*-values is (1,50) for tests with raw scores and (1,51) for tests with *z*-scores. *^∗^p*-value set to 0.01 to account for multiple comparisons.*

### Vocabulary, Visual Attention, Digit Memory

A univariate analysis of variance conducted on children’s PPVT-4 standard scores showed that participants with word reading difficulty knew significantly fewer spoken word meanings (99.4 ± 7.9) than children in the control group (110.2 ± 9.3; *F*[1,51] = 19.3, *p* < 0.001).

Overall scores for selective attention and attention switching were determined from the standard scores of eight measures of visual attention (sky search accuracy and time, attention score, map mission, creature counting accuracy and time, same and opposite world). Mean results for selective and switching attention are presented in Table 4. Univariate ANOVAs revealed a significant group difference which favored the control children on switching attention, and a smaller yet significant difference on selective attention. An additional two-way ANOVA confirmed the presence of a significant interaction between group and subtest [*F*(1,51) = 24.15, *p* < 0.001]. Children from the control group also achieved significantly higher scores than the children with word reading difficulty on digit memory forward and backward (see Table 4).

**TABLE 4:** Visual attention and digit memory skills across the groups.

**Cognitive tests**	**Control Mean (*SD*)**	**Word Reading Difficulty Mean (*SD*)**	***F*-value**	***p*-value**	**Effect size**
**Visual Attention**					
Selective Attention	9.6 (2.5)	8.1 (1.5)	6.84	0.012^∗^	0.118
Switching Attention	12.1 (2.7)	6.6 (2.2)	63.2	< 0.001^∗^	0.554
**Digit memory**					
Digit forward	12.8 (2.3)	8.6 (2.4)	41.9	< 0.001^∗^	0.451
Digit backward	12.4 (1.6)	8.0 (2.1)	73.9	< 0.001^∗^	0.592

*Univariate ANOVA *F*-values for scaled scores from the subtests of the working memory test. The df, error df for the *F*-values is (1,51). ^∗^*p*-value set to 0.01 to account for multiple comparisons.*

### Subgroup Profiles

In accordance with the second hypothesis, children were allocated to subgroups according to their pattern of comorbid deficits. To determine the individual profiles, we considered only those tasks for which norms were available. Thus, for auditory processing, FPT, DDdT, GIN and LiSN were considered. Also utilized to define the individual profiles were: the phonological processing task of elision, receptive vocabulary (PPVT), attention, and memory tasks, all of which have published norms.

In general, children attained age appropriate scores on the phonological processing task of elision, with all but a single child scoring within 1 SD of the mean or better. Similarly, all children scored within 1 SD of the mean or better on the PPVT-4 measure of receptive vocabulary. In light of this consistently good performance, the variables of phonological processing and receptive vocabulary were not included for subgrouping purposes.

**FIGURE 2:**
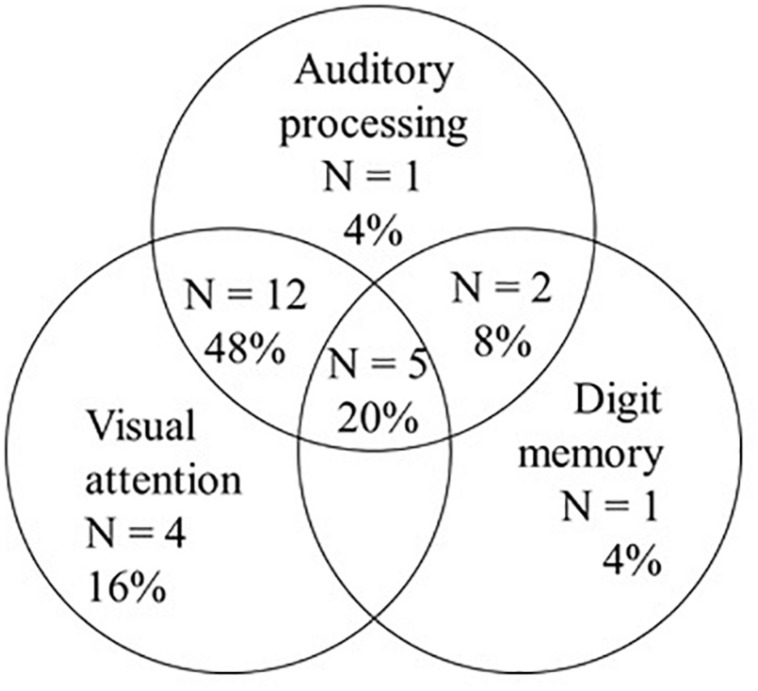
Venn diagram displaying the co-morbidities observed in the children (*n* = 25) with word reading difficulties in the current study.

Figure 2 and Table 5 show that, of the 25 children with word reading difficulties, 20% (*n* = 5) had comorbid deficits in three variables: auditory processing, visual attention, and digit memory. A larger percentage of children (56%, *n* = 14) had comorbid deficits in two variables: 12 children had auditory processing deficits and visual attention difficulties, and 2 had deficits in auditory processing and digit memory. No child experienced comorbid deficits in only visual attention and digit memory. Finally, six (24%) of the children with word reading difficulties displayed a comorbid deficit in just one other variable: four children had visual attention difficulties, one an auditory processing deficit, and one a deficit in digit memory. An alternative way of thinking about these subgrouping data is that 84% (*n* = 21) of this cohort of children with word reading difficulties had comorbid visual attention problems, and 80% had auditory processing deficits. Further detail regarding the specific deficits displayed by each child with non-word reading difficulties is presented in Table 5. This table presents the profiles of the 25 children with non-word reading deficits on word reading, auditory processing, attention, and digit memory. This table shows that, within the total cohort, children presented a tendency to have deficits on multiple auditory processing tasks (*n* = 13) or on both visual attention tasks of switching and selective (*n* = 11).

**TABLE 5:** Profiles of the 25 children with non-word reading deficits on word reading, auditory processing, attention, digit memory.

	**Age/Gender**	**Word reading [Regular/Irregular]**	**Auditory processing [FPT, DDdT (Dichotic/Diotic), GIN, LiSN-S (High and Low cue)]**	**Visual attention [Switching/Selective]**	**Backward digit memory**
*EXP17*	8/M	1.5 SD below Regular, Irregular	2 SD below FPT, DDdT and GIN	1 SD below on selective and switching	1 SD below
*EXP05*	8/M	1.5 SD below Regular	2 SD below FPT, DDdT, GIN and Low cue	2 SD below on selective and switching	1 SD below
*EXP06*	8/M	1.5 SD below Regular	2 SD below FPT, GIN, and Low cue		
*EXP25*	8.3/M	1.5 SD below Regular	2 SD below FPT and Low cue	2 SD below on selective and switching	1 SD below
*EXP88*	8.6/F	1.5 SD below Irregular, 1 SD below Regular	2 SD below on FPT and Low Cue	1 SD below on selective	
*EXP60*	8.6/F	1.5 SD below Regular, Irregular	2 SD below FPT, DDdT, and Low cue	1 SD below on selective; 2 SD below on switching	
*EXP64*	8.6/M	1.5 SD below Regular, Irregular	2 SD below FPT and GIN	1 SD below on selective and switching	
*EXP09*	8.7/F	1.5 SD below Irregular; 1 SD below Regular	2 SD below DDdT and Low cue	1 SD below on switching	
*EXP07*	8.7/M	1.5 SD below Regular, Irregular	2 SD below FPT, DDdT, and GIN	1 SD below on switching	1 SD below
*EXP16*	8.8/M	1.5 SD below Regular	2 SD below on FPT	1 SD below on switching	
*EXP91*	9.1/F	1.5 SD below Regular; 1 SD below Irregular		2 SD below on selective and switching	
*EXP33*	9.3/M	1.5 SD below Regular; 1 SD below Irregular		2 SD below on selective and switching	
*EXP73*	9.5/M	1.5 SD below Regular	2 SD below DDdT	2 SD below on selective	
*EXP46*	9.6/M	1.5 SD below Regular, Irregular	2 SD below on FPT and GIN	2 SD below on selective	
*EXP81*	9.6/F		2 SD below FPT	2 SD below on selective and switching	
*EXP29*	9.8/M	1.5 SD below Regular, Irregular	2 SD below FPT	2 SD below on selective and switching	
*EXP43*	9.8/M	1.5 SD below Regular, Irregular	2 SD below FPT;	1 SD below on selective; 2 SD below on switching	1 SD below
*EXP15*	10/M	1.5 SD below Regular, Irregular	2 SD below FPT and DDdT		1 SD below
*EXP53*	10.3/F	1.5 SD below Regular, Irregular	2 SD below DDdT	1 SD below on selective	
*EXP39*	11/M^∗^	1.5 SD below Regular, Irregular	2 SD below FPT and DDdT	2 SD below on selective and switching	
*EXP96*	11/F	1 SD below Regular			1 SD below
*EXP69*	11.1/F	1 SD below Regular	2 SD below on DDdT		1 SD below
*EXP54*	11.3/M	1.5 SD below Regular, Irregular		2 SD below on switching	
*EXP26*	11.5/F	1.5 SD below Regular, Irregular	2 SD below FPT, DDdT, GIN, and Low cue	2 SD below on selective and switching	
*EXP62*	11.7/M	1.5 SD below Regular		2 SD below on selective; 1 SD below on switching	

*All but one child had phonological processing skills to be within 1 SD while all children had vocabulary scores to be within 1 SD. ^∗^1 SD below on phonological processing task of elision.*

### Correlations Across Auditory Processing Tasks in Children With Word Reading Difficulties

Table 6 presents the Pearson’s correlation coefficients between the auditory processing measures. With age taken as covariate, Pearson correlations showed that FPT was highly correlated to GIN (*r* = −0.70, *p* < 0.001) and FD (*r* = −0.79, *p* < 0.001) but not DDdT (*r* = 0.33, *p* = 0.10). The dichotic score was correlated to the diotic score though (*r* = 0.77, *p* < 0.001). There were no more significant correlations between any of the other auditory processing measures. Furthermore, digit backwards scores were also not significantly correlated with any auditory processing measures (*p’s* > 0.05).

**TABLE 6:** Pearson’s correlation between auditory processing measures and the digit backward task.

	**Dichotic**	**Diotic**	**FPT**	**GIN**	**Low cue**	**High cue**	**FD**	**IRN**	**SAM**	**Digit backward**
Dichotic	1	0.777^∗^	0.339	−0.246	−0.335	−0.445	−0.323	0.115	−0.341	0.345
Diotic		1	0.352	−0.448	−0.245	−0.399	−0.229	0.308	−0.282	0.211
FPT			1	−0.700^∗^	−0.487	−0.424	−0.798^∗^	0.342	−0.395	0.226
GIN				1	0.386	0.371	0.521	−0.449	0.390	−0.083
Low Cue					1	0.524	0.453	−0.300	0.409	0.043
High Cue						1	0.178	−0.260	0.437	−0.139
FD							1	−0.331	0.525	−0.260
IRN								1	−0.455	0.036
SAM									1	−0.332
Digit Backward										1

*All the measures in the analysis were raw scores since standardized scores were not available for all auditory processing tasks. Therefore, age was taken as covariate for the analysis.*

Table 7 presents the Pearson’s correlation coefficients for word reading, visual attention, receptive vocabulary, and the phonological processing measure of elision. This table shows no significant correlation between selective attention and attention switching (*r* = 0.29, *p* = 0.21). This table also shows that none of the word reading measures were correlated with visual attention, receptive vocabulary, or elision (*p’s* > 0.05) Irregular word reading was, however, significantly correlated with regular word reading (*r* = 0.655, *p* < 0.001).

**TABLE 7:** Pearson correlation for word reading, visual attention, receptive vocabulary, and the phonological processing measure of elision.

	**Regular word**	**Irregular word**	**Non-word**	**Selective attention**	**Switching attention**	**PPVT**	**Elision**
Regular word reading	1	0.655^∗^	0.386	0.006	0.055	−0.192	0.302
Irregular word reading		1	0.264	−0.172	−0.130	−0.045	0.074
Non-word reading			1	−0.177	−0.071	−0.131	0.244
Selective Attention				1	0.293	−0.052	−0.306
Switching Attention					1	−0.195	0.131
PPVT						1	0.077
Elision							1

*All standardized scores were used in the analysis. Bonferroni correction was applied for the multiple comparisons in the analysis, and no variables were found to be significantly correlated to each other.*

## Discussion

The first aim of this research was to confirm the existence of average group differences between children with word reading difficulties and their peers with typical reading skills on a range of auditory processing measures. The second aim was to determine the subgroup profiles of children with word reading difficulties across a set of variables including auditory processing, visual attention, phonological processes, receptive language (vocabulary), and working memory.

Consistent with the previous literature, children with word reading difficulties performed significantly more poorly as a group on auditory processing, phonological processing (elision) receptive language (vocabulary), visual attention, and digit memory compared to children with age appropriate word reading skills. At an individual level, however, the picture is more complicated. For instance, despite significant group mean differences in receptive vocabulary and phonological awareness (elision), no individual child with a word reading difficulty scored more than 1 SD below the typical mean on vocabulary, and just one child with a reading difficulty achieved a score more than 1 SD below the mean on phonological awareness (elision). Clearly, in this case, group mean findings do not provide a reliable indicator of individual outcomes. Furthermore, individual outcomes vary markedly across variables: Some children in the current sample have relatively isolated reading problems (*n* = 6 with just one comorbid deficit), whereas others have multiple comorbid deficits of varying combinations (*n* = 19 with two or more comorbidities; see Figure 2). It is important to understand the nature of the various comorbidities to advance theoretical and clinical knowledge relating to word reading difficulties, their assessment and possible intervention.

Before considering the results further, it is important to acknowledge that the patterns of comorbidity described here depend critically on the criteria used to identify deficits. As outlined in some detail earlier in the paper (see section Performance Criteria), we adopted performance criteria that were used previously in related research literature. Regarding auditory processing, this approach meant that deficits were identified when scores fell 2 SD or more below the mean. Although the use of minus 2 SD as a criterion for atypical performance has been deemed arbitrary in the auditory processing literature (Dillon et al., 2012; Wilson and Arnott, 2013), it has the advantage of providing a conservative estimate of the occurrence of auditory processing deficits in the current cohort, which seems appropriate given our primary interest in discovering an association between auditory processing deficits and word reading difficulties. Based on previous literature, the current study employed different criteria to identify reading difficulties (−1.5 SD or more below the typical mean) and attention and memory deficits (1 SD or more below the typical mean). If these criteria were modified to −2 SD, the profiles presented here would change substantially in some respects (Table 5). For instance, none of the children would be regarded as having a digit memory deficit, and only 14 (56%) children would be considered to have visual attention deficits compared to the current 21 (84%). While this study was not designed to examine the appropriateness of the subgrouping criteria, the issue is central to how one defines co-morbidity or heterogeneity in the population of children with word reading difficulties. Future studies will need to assess the test–retest reliability of the various tasks used to measure underlying abilities and evaluate the line of demarcation between a “typical” score and an “atypical” score in standard deviation units. In the meantime, it is critical that, as researchers, we are transparent in our choices and provide clear justifications.

### Comorbidities in Children With Word Reading Difficulties

As the current results show, group effects can be misleading when they conceal marked variation in the comorbidities seen in a cohort of children with word reading difficulties. Until now, we have considered the pattern of performance across different tasks, but individual scores could also show evidence of variability according to the type of auditory processing deficits seen across individuals (Iliadou et al., 2009; Sharma et al., 2009), or the type of attention deficits, selective and/or switching. All these potential sources of individual variation could clearly be important in accounting for variability in findings across studies.

The high co-morbidities observed in the current cohort can be explained in two ways. First, there may be a causal relationship between one of the measured skills and the remaining associated skills, including word reading ability. This relationship might influence children’s competence, such that they do not perform well on one aspect, and as a result also do poorly on some or most other aspects. Some researchers have suggested that attention is the “global” deficit that guides performance across the skills (Moore et al., 2010; Snowling et al., 2018). However, this suggestion does not appear to hold true for the current cohort in which 4 out of 21 children with an attention deficit showed evidence of *no other deficit*, and a further 4 children showed evidence of deficits in auditory processing and/or digit memory, despite having *no attention deficit*. Furthermore, all except one of the 25 children with reading difficulties, including those with visual attention deficits, performed within 1 SD of the typical mean on both phonological processing (elision) and receptive vocabulary. As regards the latter finding, it remains possible that a different pattern of results might have emerged had we used a different measure of phonological processing and/or a more global measure of language ability that did not reflect vocabulary knowledge alone.

A second possible explanation for the high co-morbidities seen in the current study is that non-word reading difficulties co-exist alongside deficits in auditory processing, visual attention, and digit memory, and an altogether different skill, not measured in the current study, underpins these multiple deficits. Moore et al. (2003) raised the possibility of a perceptual learning deficit guiding performance on cognitive, reading, and auditory processing skills. Other possibilities are the effectiveness of reading instruction that children receive, and/or the amount of time that they spend engaged in reading activities. It is a limitation of the current study that time spent reading was not included in our assessment battery, which was already extensive and time-consuming. It would be useful in future studies to evaluate the association between this more practice-based variable and children’s outcomes across the range of measures used here.

### Auditory Processing Skills in Children With Word Reading Difficulty

In this study, children with word reading difficulties performed significantly more poorly, on average, on the FD, FPT, DDdT, and LiSN-S tasks compared to children with typical reading skills. These results are consistent with previous research conducted in children with word reading difficulties for FD (Halliday and Bishop, 2006a; McArthur et al., 2008; Goswami et al., 2010), FPT (Sharma et al., 2006, 2009), GIN (Zaidan and Baran, 2013), dichotic listening (Moncrieff and Black, 2007), and speech in noise percept (Bradlow et al., 2003; Ziegler et al., 2009) (which has been measured differentially across the literature).

SAM and IRN did not differ significantly across groups in the current cohort of children. This finding contrasts with that of Rocheron et al. (2002) who found a positive relationship between modulation detection, phonological processing, and reading abilities in typically developing children (Rocheron et al., 2002). However, the study included children who were severely reading-impaired (some performing 5 years below their reading age); and the deficit was measured on a passage reading task and not word reading. Therefore, it is possible that any differences in the types of auditory processing affected are a result of differences in the types of reading disorders considered in the two studies.

Table 5 provides detailed information about the various auditory processing skills affected in children with non-word reading deficits in the current study. Individual profiles show that most children had difficulty on FPT and DDdT consistent with previous research (Sharma et al., 2009). FPT is a complex task that requires children to attend to three tones that differ in frequency and are presented in a particular sequence. The children have to recognize the patterns, and label them in the correct order. The complexity of the task may be one reason for generally poor performance. In a recent study, FPT was found to be a unique predictor of word reading skills in children, which may explain why FPT is generally impacted in the current cohort of children with non-word reading difficulties (Sharma et al., 2019).

The Dichotic Digit difference task requires repetition of four numbers, and is therefore a relatively simpler task than FPT, and yet it was impacted in a similarly large number of children. It would appear, therefore, that children’s poor performance is not due solely to the complexity of the task. DDdT is a relatively new measure (Cameron et al., 2016), which includes a dichotic and a diotic listening task. The dichotic-diotic difference is able to provide a measure of dichotic advantage while accounting for cognitive contributors such as attention and memory (Cameron et al., 2016). Dichotic listening ability has been assessed previously using DDT in children with reading deficits and it was observed that children with reading disorders had deficits on DDT (Moncrieff and Black, 2007; Skarzynski et al., 2015). What is not clear is why DDT should be impacted in children with word reading deficits. Furthermore, Sharma et al. (2019) found that DDT did not contribute to word reading. Hashimoto et al. (2000) used a dichotic and diotic listening task involving phrases, and found, using functional magnetic resonance imaging, that areas such as planum temporale and superior temporal gyrus were activated more during the dichotic listening task than during the diotic task. The authors concluded that auditory areas associated with dichotic listening played a role in speech recognition. Thus, there might be an indirect relationship between dichotic listening and reading ability, with language mediating the link (Hashimoto et al., 2000). This relationship needs to be explored further, especially in light of the current study’s finding that DDdT did not correlate with FPT, thus implying that they may be measuring different aspects of auditory processing. At the same time, most of the children with word reading difficulties (*n* = 7, 32%) had both FPT and DDdT deficits.

GIN was another task where the gap threshold of seven children with word reading difficulties was higher than the expected norm. In one previous research study, a link was reported between gap detection and reading skills in children (Walker et al., 2006). More importantly, it is interesting that none of the children in the current cohort had difficulty only on GIN; they had difficulty on FPT as well. Correlations showed that FPT and GIN were highly correlated. While correlations are not indicative of a causal relationship, the associations are informative. FPT does include some level of temporal processing that GIN is also assessing. However, the control group showed only a weak to moderate correlation between FPT and GIN (*r* = −0.49, *p* = 0.01). Perhaps the association between FPT and GIN is driven by other skills along with temporal processing that would account for differences in the associations between the two tasks in the two groups. Alternatively, perhaps the weak to moderate correlation was observed because of the control group’s performance being close to ceiling on the FPT and GIN tasks (see Table 3).

The LiSN-S low cue listening situation represents the most difficult scenario wherein the target and distractors are acoustically similar and arrive from the same location. Seven children had difficulties on the low cue condition of this task. While LiSN-S has not been used previously to assess speech perception in noise in children with word reading difficulties, other similar tasks have been utilized in this population. For instance, Bradlow et al. (2003) assessed the ability of children with learning difficulties to perceive sentences (similar to LiSN-S) in noise. Consistent with the current results, the study reported that children with learning difficulties performed more poorly than their age-matched peers with typical development on the sentence perception in noise task. In another study, stepwise regression analyses showed that speech perception in noise was a unique predictor of composite word reading score (total performance across regular, irregular and non-word reading) even after phonological processing, attention, and memory were accounted for in the model (Ziegler et al., 2009). The research also reported that removal of the fine structure of speech resulted in poor speech perception similar to when noise is introduced. The authors concluded that the core deficit in children with dyslexia (reading disorder) was the lack of speech clarity that often occurs in the presence of classroom noise. This finding explains the group results in the current study and provides support for the argument that children with reading difficulties require a higher signal to noise ratio compared to their peers with typical reading skills. However, only seven children (28%) showed deficits on the low cue condition of the LiSN-S task and always with FPT deficits, yet no correlation between the tasks was observed.

In the current study, a cohort of 25 children with non-word reading difficulties participated. It is apparent that a large number of variations exist at the individual level. For instance, while all children had poor non-word reading skills, not every child had regular and irregular word reading problems. Most children with non-word reading difficulties displayed deficits on FPT, but about half showed deficits on either or both low cue of LiSN-S and GIN. It was difficult to determine which of these children would have selective and/or attention switching deficits. It was also unclear why only a third of the cohort had backward digit memory deficits. A bigger dataset collected from children who have specific non-word reading difficulties (i.e., in the absence of real word difficulties) would be useful in attempting to evaluate whether there are common subgroup profiles that can explain variability and assist in designing clinical management programs.

## Conclusion

The findings from the current study support the hypothesis that children with word reading difficulties have comorbidities across a range of skills, including auditory processing, visual attention, and digit memory. On the standardized tests of auditory processing (FPT, DDdT, LiSN-S, GIN), 80% of children with non-word reading difficulties showed a significant deficit. Although it is difficult to establish a clear link between performance on different tests, it is evident that identifying the presence of multiple deficits in individual children with reading difficulties is key to better management. One cannot assume that children with reading difficulties have a single problem. Equally important, the assumption that individual children with reading difficulties have deficits across *all* areas of functioning is also incorrect. Therefore, a multi-dimensional test battery encompassing a minimum of auditory processing, attention and memory in children with non-word reading difficulties will enable identification of important strengths and weaknesses. From a clinical perspective, the results suggest that the approach to assessment and management of children with word reading difficulties should be multidisciplinary and incorporate assessments of all relevant abilities.

## Data Availability Statement

All datasets generated for this study are included in the manuscript/Supplementary Files.

## Ethics Statement

The studies involving human participants were reviewed and approved by the Human Research Ethics Committee Macquarie University. Written informed consent to participate in this study was provided by the participants’ legal guardian/next of kin.

## Author Contributions

RG carried out the data collection. RG conducted the data analysis with inputs from MS and LC. All authors made a substantial, direct and intellectual contribution to the work, and approved it for publication.

## Conflict of Interest

The authors declare that the research was conducted in the absence of any commercial or financial relationships that could be construed as a potential conflict of interest.
