# Corrigendum: Comorbidity of auditory processing, attention, and memory in children with word reading difficulties

**DOI:** 10.3389/fpsyg.2022.1048163

**Published:** 2022-11-17

**Authors:** Rakshita Gokula, Mridula Sharma, Linda Cupples, Joaquin T. Valderrama

**Affiliations:** ^1^Department of Linguistics, Macquarie University, Sydney, NSW, Australia; ^2^HEARing Cooperative Research Centre, Melbourne, VIC, Australia; ^3^Centre for Language Sciences, Macquarie University, Sydney, NSW, Australia; ^4^National Acoustic Laboratories, Sydney, NSW, Australia

**Keywords:** word reading difficulty, auditory processing, cognition, digit memory, receptive language

In the published article, there were errors in several figures and tables, and their captions. Due to the removal of one participant, several amendments were required to the values stated. The corrected figures, tables, and captions are shown below.

Figure 2 and its corrected caption are shown below.

**Figure 2:**
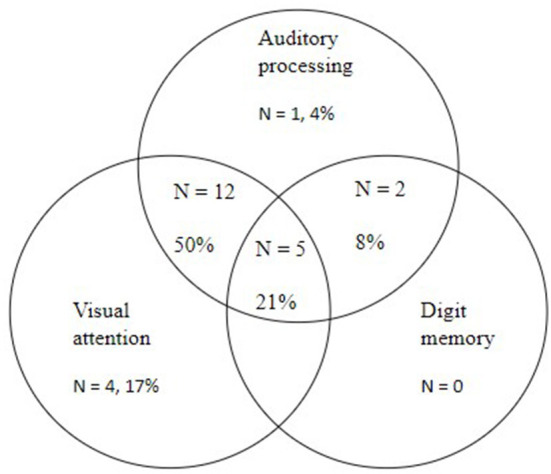
Venn diagram displaying the co-morbidities observed in the children (*n* = 24) with word reading difficulties in the current study.

Due to the removal of one participant, several corrections were also required to the text.

Corrections have been made to *Abstract*, sections *Design* and *Results*. The corrections are shown below.

*Abstract, Design*

This sentence previously stated: “Twenty-five children with word reading difficulties and 28 control children with good word reading skills participated.” The corrected sentence appears below:

“Twenty-four children with word reading difficulties and 28 control children with good word reading skills participated.”

*Abstract, Results*

This sentence previously stated “The results from children with word reading difficulties showed that 5 children (20%) had comorbid deficits in auditory processing, visual attention, and backward digit memory; whereas 12 children (48%) had comorbid auditory processing and visual attention deficits only.” The corrected sentence appears below:

“The results from children with word reading difficulties showed that 5 children (21%) had comorbid deficits in auditory processing, visual attention, and backward digit memory; whereas 12 children (50%) had comorbid auditory processing and visual attention deficits only.”

Corrections have been made to *Materials and Methods*, sections *Participants* and *Inclusion Criteria*.

*Materials and Methods, Participants*

This sentence previously stated: “Fifty-three children aged 8–11 years (Mean age in years ± SD = 9.7 ± 1.17) participated in the current study. Of the 53 children, 25 (9.5 ± 1.16 years) were identified as having reading concerns.” The corrected sentence appears below:

“Fifty-two children aged 8–11 years (Mean age in years ± SD = 9.8 ± 1.15) participated in the current study. Of the 52 children, 24 (9.5 ± 1.15 years) were identified as having reading concerns.”

*Materials and Methods, Inclusion Criteria, Paragraph 6*

This previously stated: “This table shows no significant difference for the audiometry thresholds obtained by children in the two groups from 500 to 4k Hz for both ears [*F*(3,153) = 1.64, *p* = 0.18]. The group mean audiometry result for the left and right ear is presented in Figure 1 that shows no significant difference for the extended high frequencies 8k to 12.5k Hz [*F*(4,204) = 0.35, *p* = 0.82]. The control group showed a small but significant advantage on the WNV when compared to the group with word reading difficulties [*F*(1,51) = 5.28, *p* = 0.03], but both groups scored around 1 SD above the mean on average (see Table 1).” The corrected paragraph appears below.

**Table 1:** Means (and SDs in parentheses) for age, PTA (0.5–4 kHz), and WNV scores for children in the two groups.

**Groups**	Age*(SD)	PTA**Right (SD)	PTA**Left (SD)	**WNV (SD)**
Control, *N* = 28	10.0 (1.1)	4.4 (3.4)	3.2 (3.1)	118.7 (10.4)
Females, *N* = 11	9.3 (0.9)	4.2 (3.9)	3.4 (3.3)	119.2 (10.6)
Males, *N* = 17	10.5 (1.0)	4.6 (3.2)	3.0 (3.0)	118.4 (10.5)
Word reading difficulty, *N* = 24	9.5 (1.2)	6.1 (4.5)	5.4 (3.8)	112.4 (9.5)
Females, *N* = 8	9.8 (1.1)	5.2 (4.3)	4.6 (4.8)	113.0 (5.3)
Males, *N* = 16	9.4 (1.2)	6.3 (4.7)	5.5 (3.3)	115.3 (11.1)

^*^Age is presented in years. ^**^PTA is presented in dB HL.

“Table 1 presents the means and SDs of children's age, PTA, and WNV scores according to group. This table shows no significant difference for the audiometry thresholds obtained by children in the two groups from 500 to 4k Hz for both ears [*F*(3, 147) = 0.29, *p* = 0.84]. The group mean audiometry result for the left and right ear is presented in Figure 1 that shows no significant difference for the extended high frequencies 8k to 12.5k Hz [*F*(4, 196) = 0.79, *p* = 0.54]. Both groups scored similarly and around 1 SD above the mean on average on WNV [*F*(1, 50) = 2.94, *p* = 0.09].”

Corrections have been made to several sections within *Results*.

*Results, Tests of Reading and Phonological Processing*

The paragraph previously stated: “By contrast, the group with word reading difficulties had mean z-scores of −1.9 (SD = 0.40), −1.4 (SD = 0.66), and −1.9 (SD = 0.40) respectively. Children in the control group achieved standard scores of 13.8 (SD = 1.20) on the phonological awareness test of elision while the children with word reading difficulties had an average standard score of 10.3 (SD = 2.65) with a statistically significant difference between the groups (*F*[1,51] = 45.0, *p* < 0.001).” The corrected text appears below.

“By contrast, the group with word reading difficulties had mean z-scores of −1.9 (SD = 0.42), −1.5 (SD = 0.56), and −1.9 (SD = 0.43) respectively. Children in the control group achieved standard scores of 13.8 (SD = 1.20) on the phonological awareness test of elision while the children with word reading difficulties had an average standard score of 10.3 (SD = 2.71) with a statistically significant difference between the groups [*F*(1, 50) = 38.9, *p* < 0.001].”

*Results, Vocabulary, Visual attention, Digit memory, Paragraph 1*

The sentence previously stated: “A univariate analysis of variance conducted on children's PPVT-4 standard scores showed that participants with word reading difficulty knew significantly fewer spoken word meanings (99.4 ± 7.9) than children in the control group (110.2 ± 9.3; *F*[1,51] = 19.3, *p* < 0.001).” The corrected sentence appears below.

“A univariate analysis of variance conducted on children's PPVT-4 standard scores showed that participants with word reading difficulty knew significantly fewer spoken word meanings (100.0 ± 7.88) than children in the control group [110.3 ± 9.3; *F*(1, 50) = 18.0, *p* < 0.001].”

*Results, Vocabulary, Visual attention, Digit memory, Paragraph 2*

The sentence previously stated “An additional two-way ANOVA confirmed the presence of a significant interaction between group and subtest [*F*(1,51) = 24.15, *p* < 0.001].” The corrected sentence appears below.

“An additional two-way ANOVA confirmed the presence of a significant interaction between group and subtest [*F*(1, 50) = 19.74, *p* < 0.001].”

*Results, Subgroup Profiles, Paragraph 3*

The paragraph previously stated: “Figure 2 and Table 5 show that, of the 25 children with word reading difficulties, 20% (*n* = 5) had comorbid deficits in three variables: auditory processing, visual attention, and digit memory. A larger percentage of children (56%, *n* = 14) had comorbid deficits in two variables: 12 children had auditory processing deficits and visual attention difficulties, and 2 had deficits in auditory processing and digit memory. No child experienced comorbid deficits in only visual attention and digit memory. Finally, six (24%) of the children with word reading difficulties displayed a comorbid deficit in just one other variable: four children had visual attention difficulties, one an auditory processing deficit, and one a deficit in digit memory. An alternative way of thinking about these subgrouping data is that 84% (*n* = 21) of this cohort of children with word reading difficulties had comorbid visual attention problems, and 80% had auditory processing deficits. Further detail regarding the specific deficits displayed by each child with non-word reading difficulties is presented in Table 5. This table presents the profiles of the 25 children with non-word reading deficits on word reading, auditory processing, attention, and digit memory. This table shows that, within the total cohort, children presented a tendency to have deficits on multiple auditory processing tasks (n = 13) or on both visual attention tasks of switching and selective (*n* = 11).” The corrected paragraph appears below.

**Table 3:** Univariate ANOVA results alongside the means (and standard deviations in parentheses) across the two groups for the individual auditory processing tests.

**Test**		**Control mean (SD)**	**Word reading difficulty mean (SD)**	***F*-value**	***p*-value**	**Effect size**
FD	Run 1 (log)	1.09 (0.31)	1.76 (0.53)	27.79	< 0.001^*^	0.362
	Run 2 (log)	1.00 (0.30)	1.63 (0.58)	21.54	< 0.001^*^	0.305
IRN	32 iterations	19.20 (2.81)	18.61 (2.69)	0.42	0.522	0.008
	04 iterations	13.28 (3.10)	11.81 (1.96)	4.45	0.040	0.083
SAM	40 Hz	−15.79 (1.60)	−14.36 (3.49)	2.29	0.137	0.045
	4 Hz	−11.96 (2.57)	−9.15 (3.58)	7.70	0.008	0.136
FPT	Right (%)	93.79 (6.96)	69.41 (22.11)	25.49	< 0.001^*^	0.342
	Left (%)	92.24 (11.12)	66.92 (24.14)	20.56	< 0.001^*^	0.296
GIN	Right (ms)	4.96 (0.88)	6.46 (1.91)	9.93	0.003	0.168
	Left (ms)	5.14 (0.80)	6.46 (1.91)	7.93	0.007	0.139
DDdT^#^	Dichotic (z-score)	0.63 (1.15)	−1.02 (1.19)	25.70	< 0.001^*^	0.339
	Diotic (z-score)	0.59 (0.89)	−1.23 (1.26)	37.35	< 0.001^*^	0.428
LiSN– S^#^	Low cue score (z-score)	−0.43 (0.81)	−1.26 (1.00)	10.71	0.002	0.176
	High cue score (z-score)	0.43 (0.75)	−0.37 (0.88)	12.41	0.001	0.199
	Talker advantage (z-score)	−0.18 (0.88)	−0.33 (0.98)	0.32	0.573	0.006
	Spatial advantage (z-score)	0.02 (1.27)	−0.42 (1.0)	1.90	0.175	0.037
	Total advantage (z-score)	0.74 (0.79)	0.21 (0.84)	5.33	0.025	0.096

In these analyses, the df, error df for the F-values is (1, 49) for tests with raw scores and ^#^ (1, 50) for tests with z-scores. *p-value < 0.001 to account for multiple comparisons.

**Table 4:** Univariate ANOVA results alongside the means (and standard deviations in parentheses) across the two groups for the visual attention and working memory tests.

**Cognitive tests**	**Control mean (SD)**	**Word reading difficulty mean (SD)**	***F*-value**	***p*-value**	**Effect size**
**Visual attention**
Selective attention	9.6 (2.5)	7.8 (1.6)	8.15	0.006	0.143
Switching attention	12.1 (2.7)	6.6 (2.2)	55.9	< 0.001^*^	0.533
**Digit memory**
Digit forward	12.8 (2.4)	8.7 (2.5)	33.9	< 0.001^*^	0.409
Digit backward	12.4 (1.6)	8.0 (2.1)	64.8	< 0.001^*^	0.569

The df, error df for the F-values is (1, 50). *p-value < 0.001 to account for multiple comparisons.

**Table 5:** Profiles of the 24 children with non-word reading deficits on word reading, auditory processing, attention, digit memory.

	**Age/Gender**	**Word reading [Regular/Irregular]**	**Auditory processing [FPT, DDdT (Dichotic/Diotic), GIN, LiSN-S (High & Low cue)]**	**Visual attention [Switching/Selective]**	**Backward digit memory**
EXP17	8/M	1.5SD below Regular, Irregular	2SD below FPT, DDdT, and GIN	1SD below on selective and switching	1SD below
EXP05	8/M	1.5SD below Regular	2SD below FPT, DDdT, GIN, and Low cue	2SD below on selective and switching	1SD below
EXP06	8/M	1.5SD below Regular	2SD below FPT, GIN, and Low cue		
EXP25	8.3/M	1.5SD below Regular	2SD below FPT and Low cue	2SD below on selective and switching	1SD below
EXP88	8.6/F	1.5SD below Irregular, 1SD below Regular	2SD below on FPT and Low Cue	1SD below on selective	
EXP60	8.6/F	1.5SD below Regular, Irregular	2SD below FPT, DDdT, and Low cue	1SD below on selective; 2SD below on switching	
EXP64	8.6/M	1.5SD below Regular, Irregular	2SD below FPT and GIN	1SD below on selective and switching	
EXP09	8.7/F	1.5SD below Irregular; 1SD below Regular	2SD below DDdT and Low cue	1SD below on switching	
EXP07	8.7/M	1.5SD below Regular, Irregular	2SD below FPT, DDdT, and GIN	1SD below on switching	1SD below
EXP16	8.8/M	1.5SD below Regular	2SD below on FPT	1SD below on switching	
EXP91	9.1/F	1.5SD below Regular; 1SD below Irregular		2SD below on selective and switching	
EXP33	9.3/M	1.5SD below Regular; 1SD below Irregular		2SD below on selective and switching	
EXP73	9.5/M	1.5SD below Regular	2SD below DDdT	2SD below on selective	
EXP46	9.6/M	1.5SD below Regular, Irregular	2SD below on FPT and GIN	2SD below on selective	
EXP81	9.6/F		2SD below FPT	2SD below on selective and switching	
EXP29	9.8/M	1.5SD below Regular, Irregular	2SD below FPT	2SD below on selective and switching	
EXP43	9.8/M	1.5SD below Regular, Irregular	2SD below FPT;	1SD below on selective; 2SD below on switching	1SD below
EXP15	10/M	1.5SD below Regular, Irregular	2SD below FPT and DDdT		1SD below
EXP53	10.3/F	1.5SD below Regular, Irregular	2SD below DDdT	1SD below on selective	
EXP39	11/M*	1.5SD below Regular, Irregular	2SD below FPT and DDdT	2SD below on selective and switching	
EXP69	11.1/F	1SD below Regular	2SD below on DDdT		1SD below
EXP54	11.3/M	1.5SD below Regular, Irregular		2SD below on switching	
EXP26	11.5/F	1.5SD below Regular, Irregular	2SD below FPT, DDdT, GIN, and Low cue	2SD below on selective and switching	
EXP62	11.7/M	1.5SD below Regular		2SD below on selective; 1SD below on switching	

All but one child had phonological processing skills to be within 1SD while all children had vocabulary scores to be within 1SD. ^*^1 SD below on phonological processing.

“Figure 2 and Table 5 show that, of the 24 children with word reading difficulties, 21% (*n* = 5) had comorbid deficits in three variables: auditory processing, visual attention, and digit memory. A larger percentage of children (58%, *n* = 14) had comorbid deficits in two variables: 12 children had auditory processing deficits and visual attention difficulties, and 2 had deficits in auditory processing and digit memory. No child experienced comorbid deficits in only visual attention and digit memory. Finally, six (25%) of the children with word reading difficulties displayed a comorbid deficit in just one other variable: 4 children had visual attention difficulties, and 1 an auditory processing deficit. An alternative way of thinking about these subgrouping data is that 88% (*n* = 21) of this cohort of children with word reading difficulties had comorbid visual attention problems, and 83% had auditory processing deficits. Further detail regarding the specific deficits displayed by each child with non-word reading difficulties is presented in Table 5. This table presents the profiles of the 24 children with nonword reading deficits on word reading, auditory processing, attention, and digit memory. This table shows that, within the total cohort, children presented a tendency to have deficits on multiple auditory processing tasks (*n* = 13) or on both visual attention tasks of switching and selective (*n* = 11).”

*Results, Correlations Across Auditory Processing Tasks in Children With Word Reading Difficulties, Paragraph 1*

The paragraph previously said, “Table 6 presents the Pearson's correlation coefficients between the auditory processing measures. With age taken as covariate, Pearson correlations showed that FPT was highly correlated to GIN (*r* = −0.70, *p* < 0.001) and FD (*r* = −0.79, *p* < 0.001) but not DDdT (*r* = 0.33, *p* = 0.10). The dichotic score was correlated to the diotic score though (*r* = 0.77, *p* < 0.001). There were no more significant correlations between any of the other auditory processing measures. Furthermore, digit backwards scores were also not significantly correlated with any auditory processing measures (*p's* > 0.05).”

**Table 6:** Pearson's correlation between auditory processing measures and the digit backward task.

	**Dichotic**	**Diotic**	**FPT**	**GIN**	**Low Cue**	**High cue**	**FD**	**IRN**	**SAM**	**Digit backward**
Dichotic	1	0.854*	0.499*	−0.440	−0.416	−0.282	−0.477*	0.089	−0.148	0.569*
Diotic		1	0.540*	−0.496	−0.419	−0.191	−0.481*	0.157	−0.144	0.561*
FPT			1	−0.795*	−0.398	−0.205	−0.680*	0.468	−0.416	0.477*
GIN				1	0.382	0.297	0.598*	−0.344	0.258	−0.354
Low cue					1	0.056	0.459	−0.016	−0.020	−0.364
High cue						1	0.126	−0.121	0.088	−0.168
FD							1	−0.075	0.009	**−0.473**
IRN								1	**−0.843**	0.127
SAM									1	−0.132
Digit backward										1

All the measures in the analysis were raw scores since standardized scores were not available for all auditory processing tasks. Therefore, age was taken as covariate. Bold values and * indicates *p* ≤ 0.001.

**Table 7:** Pearson correlation for word reading, visual attention, receptive vocabulary, and the phonological processing measure of elision. Standardized scores were used in the analysis.

	**Regular word**	**Irregular**	**Non-**	**Selective**	**Switching**	**PPVT**	**Elision**
	**word**	**word**	**attention**	**attention**	**standard**	
Regular word reading	1	0.835*	0.916*	0.441	0.792*	0.493*	0.650*
Irregular word reading		1	0.855*	0.315	0.633*	0.529*	0.590*
Non-word reading			1	0.394	0.729*	0.456*	0.678*
Selective Attention				1	0.456	0.389	0.136
Switching Attention					1	0.337	0.550*
PPVT						1	0.403
Elision							1

Bonferroni correction was applied for multiple comparisons in the analysis, and some variables were found to be significantly correlated to each other. Bold values and * indicates *p* ≤ 0.001.

The corrected paragraph appears below.

“Table 6 presents the Pearson's correlation coefficients between the auditory processing measures. With age taken as covariate, Pearson correlations showed that FPT was highly correlated to GIN (*r* = −0.75, *p* < 0.001) and FD (*r* = −0.73, *p* < 0.001) but not to combined score of DDdT (*r* = −0.19, *p* = 0.19). The dichotic score was correlated to the diotic score (*r* = 0.85, *p* < 0.001). Digit backwards scores were significantly correlated to FPT, and FD (*p's* ≤ 0.001).”

*Results, Correlations Across Auditory Processing Tasks in Children With Word Reading Difficulties, Paragraph 2*

The sentence previously said, “This table shows no significant correlation between selective attention and attention switching (*r* = 0.29, *p* = 0.21). This table also shows that none of the word reading measures were correlated with visual attention, receptive vocabulary, or elision (*p's* > 0.05) Irregular word reading was, however, significantly correlated with regular word reading (*r* = 0.655, *p* < 0.001).” The corrected sentence appears below.

“This table shows significant correlation between selective attention and attention switching (*r* = 0.49, *p* < 0.001). This table also shows that the word reading measures were significantly correlated to each other and to switching attention, receptive vocabulary, and elision (*p* ≤ 0.001).”

Corrections have also been made to section *Discussion*

*Discussion, Paragraph 3*

The sentence previously said, “For instance, none of the children would be regarded as having a digit memory deficit, and only 14 (56%) children would be considered to have visual attention deficits compared to the current 21 (84%).” The corrected sentence appears below.

“For instance, none of the children would be regarded as having a digit memory deficit, and only 14 (58%) children would be considered to have visual attention deficits compared to the current 21 (88%).”

*Discussion, Comorbidities in Children With Word Reading Difficulties, Paragraph 2*

The sentence previously said, “However, this suggestion does not appear to hold true for the current cohort in which 4 out of 21 children with an attention deficit showed evidence of *no other deficit*, and a further 4 children showed evidence of deficits in auditory processing and/or digit memory, despite having *no attention deficit*. Furthermore, all except one of the 25 children with reading difficulties, including those with visual attention deficits, performed within 1 SD of the typical mean on both phonological processing (elision) and receptive vocabulary.” The corrected sentence appears below.

“However, this suggestion does not appear to hold true for the current cohort in which 4 out of 21 children with an attention deficit showed evidence of *no other deficit*, and a further 3 children showed evidence of deficits in digit memory and/or auditory processing, despite having *no attention deficit*. Furthermore, all except one of the 24 children with reading difficulties, including those with visual attention deficits, performed within 1SD of the typical mean on both phonological processing (elision) and receptive vocabulary.”

*Discussion, Auditory Processing Skills in Children With Word Reading Difficulty, Paragraph 4*

The sentence previously said, “At the same time, most of the children with word reading difficulties (*n* = 7, 32%) had both FPT and DDdT deficits.” The corrected sentence appears below.

“At the same time, only a third of the children with word reading difficulties (*n* = 7, 29%) had both FPT and DDdT deficits.”

*Discussion, Auditory Processing Skills in Children With Word Reading Difficulty, Paragraph 7*

The sentence previously said, “In the current study, a cohort of 25 children with non-word reading difficulties participated.” The corrected sentence appears below.

“In the current study, a cohort of 24 children with non-reading word difficulties participated.”

Corrections have also been made to section *Conclusion*

The sentence previously said, “On the standardized tests of auditory processing (FPT, DDdT, LiSN-S, GIN), 80% of children with non-word reading difficulties showed a significant deficit.” The corrected sentence appears below.

“On the standardized tests of auditory processing (FPT, DDdT, LiSN-S, GIN), 83% of children with non-word reading difficulties showed a significant deficit.”

The authors apologize for these errors and state that they do not change the scientific conclusions of the article in any way. The original article has been updated.

## Publisher's note

All claims expressed in this article are solely those of the authors and do not necessarily represent those of their affiliated organizations, or those of the publisher, the editors and the reviewers. Any product that may be evaluated in this article, or claim that may be made by its manufacturer, is not guaranteed or endorsed by the publisher.

