# Supplementary material for: Comorbidity of Auditory Processing, Attention, and Memory in Children With Word Reading Difficulties
Source: Front Psychol. 2019 Oct 22;10:2383. doi: 10.3389/fpsyg.2019.02383 (PMC6817942; doi:10.3389/fpsyg.2019.02383)
Supplement: Supplementary file 1 [file Table_1.pdf]

Supplementary Table 1: Details of the reading and auditory processing tests employed in the study.

Measures	Tests	Procedure
**Reading ability**	Castles and Coltheart 2 (CC2)	*Stimuli:* The CC2 was used to assess oral reading accuracy for regular (e.g. take), irregular (e.g. eye), and non-words (e.g. gop) (Castles et al. 2009). The test includes three lists of 40 items presented for the children to read out loud. The words were presented in an intermixed fashion on 120 x 85cm sized cards.<br>*Procedure:* Words from the three lists were presented in a predetermined order. The presentation was randomised such that no child could predict which list the next word came from.<br>*Response and Scoring:* A score of 1 was allocated for each word read correctly. When a participant read five consecutive words from a list incorrectly, presentation of words from that list ceased. Testing ceased when a participant had responded incorrectly to five consecutive words in each of the three lists. The test results included raw scores and z scores obtained by a child. The scores were distributed according to age. The children with word reading difficulties were identified based on a z-score of at least 1.5 SD below the typical mean on all three-word types (i.e., regular words, irregular words, and non-words). A higher score (in terms of words read correctly) represented better performance in the test.
**Phonological Processing**	Comprehensive Test of Phonological Processing (CTOPP)	*Stimuli:* The elision subtext from within the CTOPP was used to measure the phonological processing skill (Wagner et al., 1999). The task begins with three practice trials after which 20 stimuli are presented.<br>*Procedure:* The test includes presentation of words to children who are then asked to remove a sound from the word and produce the resultant real word. For example, 'tiger' without the 'g' is 'tire'.<br>*Response and Scoring:* A score of 1 is provided for each correct response. The presentation of words is ceased when a child responds incorrectly to three consecutive words. The test includes raw score and a standard score for a child, distributed according to age. A higher score represented better performance in the test.
	Frequency Pattern Test (FPT)	*Stimuli:* Three tones were presented one after the other. The "high" tone had a frequency of 1100 Hz, and the "low" tone had a frequency of 880 Hz (Musiek 1994). The tones were presented through an Audiometer, which received the input from a Laptop. Thirty stimuli were presented per ear.<br>*Procedure:* The participants had to convey the pitch of the three tones in a set by labelling them as being high or low.<br>*Response and Scoring:* The verbal responses of the participants were marked, with each correct response yielding 1 point. The score out of 30 was converted into a percentage score. The norms for the

	test were taken from Cameron and Dillon (2005). A higher score (in terms of sequences identified) represented better frequency patterning ability in a child.
Gaps in Noise (GIN)	*Stimuli:* White noise containing gaps of differing lengths was played through an Audiometer which received the input from a Laptop. The level of the stimulus was set to 50 dB HL so that it replicated comfortable listening loudness. The white noise was 500 ms in length. The length of the gaps embedded in the noise could be 20, 12, 10, 8, 6, 5, 4, 3, or 2 ms (Shinn et al. 2009). Each set of stimuli could have 0, 1, 2 or 3 gaps. Each of the 9 gap lengths was presented 6 times in the 30 stimuli presented per ear.<br>*Procedure:* The participants were asked to count the gaps in a particular stimulus set.<br>*Response and Scoring:* The responses of the children were marked on a scoring sheet which displayed the length of the gap identified by the participant. A lower score (in ms) represented better response in terms of length of gap identified by a child.
Dichotic Digit Difference Test (DDdT)	*Stimuli:* The DDdT (Cameron et al. 2016) created by the National Acoustic Laboratories (NAL) was used. Digits were presented to both ears simultaneously. Two subtests: Free Recall (Dichotic) and Diotic task were used in the study. In the dichotic task, the children heard two different stimuli in the two ears. In the Diotic task, the same four digits were presented in both ears.<br>*Procedure:* The participants were asked to repeat the numbers they heard.<br>*Response and Scoring:* The responses of the children were input into software which calculated their scores with each response. The percentage correct score is the final score to be compared with the normative scores. The norms for the test were taken from Cameron et al. (2016). A higher score (in percentage) represented better dichotic and diotic listening in a child.
Listening in Spatialized Noise-Sentences (LiSN-S)	*Stimuli:* The LiSN-S test (Cameron et al. 2006) which was developed at the NAL and is commercially available was used. Target sentences were present amidst distracting sentences. The distractors were presented differently in four different subtests: a) distractor speaker different from the target speaker presented from 90° azimuth; b) distractor speaker same as the target speaker presented from 90° azimuth; c) distractor speaker different from the target speaker presented from 0° azimuth; and d) distractor speaker same as the target speaker presented from 0° azimuth.<br>*Procedure:* The participants were asked to repeat the target sentences, which were presented after a ‘beep,’ and to ignore the distractor sentences.<br>*Response and Scoring:* The target stimulus level was adaptively varied by the software to estimate the speech reception threshold (SRT) for the subtests. The levels were adjusted in 4 dB steps until the first reversal in performance was recorded and in 2 dB steps thereafter. The norms for the test were provided automatically by the software (Cameron & Dillon 2008).

Supplementary Table 2. Details of the visual attention, digit memory and vocabulary tests employed in the study.

Measures	Tests	Procedure
**Visual Attention**	Test of Everyday Attention for Children (TEA-Ch): Selective Attention	*Stimuli:* The TEA-Ch (Manly et al. 2001) was used to measure selective attention and attention switching abilities in children. In the Sky Search subtest, participants were provided with an A3 size sheet containing pictures. The participants were instructed that the pictures represented spaceships that were in pairs. The Map Mission task included an A3 sheet with a map on it.<br>*Procedure:* The participants had to find the pairs where both the spaceships were the same. The time taken to do this was recorded. The test finished when the child ticked the box at the bottom, indicating that they had found the spaceships. In the map mission task, the aim was for the children to find as many restaurants (marked by a pair of fork and knife) on a map as they could within 1 minute.<br>*Response and Scoring:* For the sky search task, the time taken for a participant to find the spaceships was noted. The time taken to find the targets divided by the number of targets found provided us with the raw score for the subtest. The same was then converted to a scaled score as per the age of the child.<br>For the map mission task, the number of targets found in 1-minute yielded the raw score. The same was then converted into a scaled score as per the age of the child.
	Test of Everyday Attention for Children: Attention Switching	*Stimuli:* The Creature Counting subtest consisted of creatures on a page. Arrows pointing upwards or downwards were placed between the creatures. The upward pointing arrow guided the counting to be continued conventionally. The downward pointing arrow asked for counting to be continued in the reverse order.<br>The Opposite World subtest included the numbers 1 and 2 on a page. The numbers were present in a random sequence. In the same world subtest, the numbers were read as they are. In the opposite world subtest, the number '1' was read as two and '2' as one. Two sets of 24-stimuli tasks were conducted for each subtest.<br>*Procedure:* The participants were asked to modify the counting technique depending on the arrows placed between the creatures. The time taken by a child to count all the creatures on a page was measured. In the Opposite World subtest, the time taken by the child to read all the numbers on a page was the score for the task.<br>*Response and Scoring:* The time taken for the creatures to be counted divided by the correct change in the counting direction yielded the raw score for the task. The same was converted into

		a scaled score as per the age of the child.<br>The time taken for the digits to be read correctly yielded the raw score for the opposite world task. The same was converted into a scaled score with respect to the age of the child.
**Digit memory**	Clinical Evaluation of Language Fundamentals fourth edition (CELF-4), Australian standardised edition	*Stimuli:* The digit forwards and backwards subtest of CELF-4 (Semel et al. 2006) was used to measure digit memory. The stimulus list contained digits in increasing order of difficulty, beginning with two digits and ceasing at a length of 8 digits. Two sets of digits were presented at each digit length. The participant was presented with the digits once only, using live voice. If the participant responded correctly to only one of the two sets within a digit length, testing continued. The test was discontinued once the subject scored 0 on both parts of an item.<br>*Procedure:* The participant was asked to repeat the digits as heard, or in a backward fashion (e.g. Stimulus = 4 9 1; Response = 1 9 4).<br>*Response and Scoring:* Each correct response of the participant yields a score of 1. A total of the responses of the participant per list yields the raw score for the list. The same may then be converted into a scaled score according to the age of the child. Higher score (in terms of digits repeated) represented better digit memory in a child.
**Receptive vocabulary**	Peabody Picture Vocabulary Test 4th Edition (PPVT 4)	*Stimuli:* The PPVT 4 is a test of receptive vocabulary (Dunn & Dunn 2007). A picture book (set up like an easel of 8.5 x 11 inches) with four pictures on each page was presented to the participant, who was required to point to the picture depicting the word spoken by the experimenter. The test had a list of words for each age range. The basal set was identified as the set of pictures for an age range wherein the participant made no more than one mistake out of a set of 12 per age range. The ceiling set was the set wherein the participant made eight errors or more within 12 test items per age group, wherein the testing ceased.<br>*Procedure:* The participant was presented with a word (live voice) and had to point to the corresponding picture.<br>*Response and Scoring:* Each correct response yielded a score of 1. The difference between the last item score and the number of errors provided the raw score for the participant. The same was then converted into a scaled score according to the age of the child. A higher score (in terms of the pictures for word identified) represented better receptive vocabulary of a child.
